# Plant–soil feedback responses of four dryland crop species under greenhouse conditions

**DOI:** 10.1002/pei3.10035

**Published:** 2020-12-07

**Authors:** Knowledge Mushonga, Joachim M. Steyn, Wijnand J. Swart, Jacquie E. van der Waals

**Affiliations:** ^1^ Department of Plant and Soil Sciences, Forestry and Agricultural Biotechnology Institute (FABI) University of Pretoria Hatfield South Africa; ^2^ Department of Applied Biology and Biochemistry National University of Science and Technology Ascot, Bulawayo Zimbabwe; ^3^ Department of Plant and Soil Sciences University of Pretoria Hatfield, Pretoria South Africa; ^4^ Department of Plant Sciences University of the Free State Bloemfontein South Africa

**Keywords:** crop rotation, monoculture, plant performance, soil conditioning, soil legacies

## Abstract

Plant–soil feedbacks (PSFs) give a mechanistic understanding on how soil properties established by previous plant species go on to influence the performance of the same or different species in monoculture, intercropping or crop rotation systems. We hypothesized that different dryland crops such as *Zea mays* L., *Helianthus annuus* L., *Phaseolus vulgaris* L., and *Glycine max* L. (Merr.) will have soil legacies that are related to the crop type. We used a two‐phase experiment to test plant performance in soils previously cultivated with the same or different plant species under greenhouse conditions. The positive plant growth for all species in their own soil microbiota suggests that mutualists had a greater impact on plant performance than pathogens. The consistent positive soil–feedback results of *P. vulgaris* were strongly associated with their own beneficial soil microbiota, meaning that the conditioning phase legacy of mutualists and decomposers were more significant than pathogens under monoculture. Despite successful nodulation in sterilized and inoculated soils, *G. max* unexpectedly showed neutral and insignificant positive plant feedbacks, respectively. *Helianthus annuus* was superior to other crop species in creating active carbon stocks and an enzymatically active soil for the next crop. Microbial biomass results suggest that raising fungal relative to bacterial biomass can be achieved by increasing the frequency of *H. annuus* in rotation sequences. However, more studies are necessary to evaluate whether these elevated ratios promote or depress plant performance under field conditions. This study showed that relative to other dryland crops, *H. annuus* seems to have the potential of increasing fungal to bacterial ratios, raising legacies in active carbon stocks and soil microbial activity that may be crucial to successional planting in dryland systems.

## 1 INTRODUCTION

Plant–soil feedback (PSF) represents plant performance linked to soil legacies established earlier on by the same or other plant species (Kulmatiski & Kardol, 2008). A positive feedback occurs when subsequent plant growth increases, whereas in a negative feedback, growth is retarded. The direction (sign) and strength of PSFs are determined by the net effects of the associations between mutualists, pathogens and plants (Mariotte et al., 2018). Soil conditions cultivated by ‘self’ or ‘own’ plant species are known as conspecific or ‘home’ soils, while those cultivated by ‘other’ or ‘foreign’ plant species are referred to as heterospecific or ‘away’ soils (Forero et al., 2019). In this study, we hypothesized that the phytometer species represent plant growth in the feedback/test phase as a result of changes and balance of soil organisms such as pathogens and mutualists, which cause a decrease and an increase in successive plant growth, respectively (Brinkman et al., 2010). The objective of this study was to determine how plant biomass responds to temporal changes in selected soil properties (biological, chemical and physical) induced in succession planting over time using a mechanistic PSF experimental approach, as proposed by Dias et al. (2014).

We used medium‐maturity plant species to minimize confounding differences common to fast‐ versus slow‐growing plants in feedback experiments (De Long et al., 2019). We expected that a cereal grass (*Zea mays* L.), nitrogen‐fixing leguminous forbs (*Phaseolus vulgaris* L. and *Glycine max* (Merr.)) and a non‐leguminous forb (*Helianthus annuus* L.) will have soil legacies that are linked to the plant type and functional groups. The legumes build nitrogen legacies in the soil that drive positive PSF, whereas *H. annuus* and *Z. mays* are net consumers of nitrogen that drive negative PSF (Cesarano et al., 2017; Cortois et al., 2016).

The nitrogen requirement of *Z. mays* is approximately twice that of *H. annuus*, *P. vulgaris* and *G. max* in dryland agroecosystems (FERTASA, 2016). Moreover, the root architecture and low cation exchange capacity of monocots such as *Z. mays* promote a soil legacy poor in monovalent cations such as sodium and ammonium ions, whereas dicot legumes (*P. vulgaris* and *G. max*) impoverish the soil of divalent cations such as calcium and magnesium ions (Fageria, 2013). The less dense and extensive tap root system of *H. annuus* allows it to efficiently draw out solutes and water from the soil. Nevertheless, these species are strategic crops under dryland production systems and are cultivated in rotation systems to maximize profitability, improve crop yields, and break pest and disease cycles under rain‐fed conditions (Montgomery et al., 2017; Nel, 2005).

Bardgett and McAlister (1999) demonstrated that unfertilized (nutrient‐poor) grassland soils supported a higher fungal to bacterial biomass ratio than disturbed and fertile (nutrient‐rich) grassland soils. The soil environment under the influence of plant roots, that is the rhizosphere, is a microenvironment which supports more soil microbial growth and maintenance than loose field soil or bulk soil (Bardgett, 2009). The influence of root activity on soil microbial communities and nutrient legacies varies in terms of the quality and quantity of the exudates they produce, which subsequently promotes nutrient availability, sustains mutualists and reduces stress (Canarini et al., 2019; Ryan et al., 2009). Other abiotic changes such as soil pH and the accumulation of allelochemicals have an impact on microbial composition and biomass (Cesarano et al., 2017). Moreover, drought conditions also disrupt plant–soil interactions and Fry et al. (2018) found that drought led to neutral PSFs on two grassland forbs.

The knowledge of feedback mechanisms is crucial to successional planting in dryland cropping systems such as crop rotations or monoculture because of the differences in plant species and genotypes. *Zea mays* thrives best in grassland biomes of South Africa, but *H. annuus* performs better than *Z. mays* on alkaline sandy soils (Smith, 2016). Understanding plant–soil feedbacks provides insight into the flow‐on impact of historical soil properties on the performance of the succeeding plant species (Kos et al., 2015). In this study, soil legacies for microbial biomass were estimated using phospholipid fatty acid analysis (PLFA), while for active carbon and soil enzyme activity, the permanganate‐oxidizable active carbon method and the fluorescein diacetate (FDA) method were used, respectively. Phospholipid fatty acid analysis (PLFAs) is a biochemical technique that gives fungal to bacterial biomass ratio and active biomass of microbes (Frostegård & Bååth, 1996; Ladygina & Hedlund, 2010; Tavi et al., 2013). The patterns and swinging ratios of these lipid biomarkers in soils have been linked to nutrient cycling, organic matter decay and carbon sequestration (Willers et al., 2015).

Active carbon is readily available as carbon and energy source for soil microbial community food webs. It is very sensitive to management effects and more closely linked to biologically mediated soil properties such as respiration, microbial biomass and aggregation than measurements of soil organic carbon (Preusser et al., 2019; Weil et al., 2003).

FDA analysis quantifies the microbial activity based on enzyme activity linked to microorganisms present in the soil (Schumacher et al., 2015). Soil protease activity increases under limited nitrogen (Bardgett & Wardle, 2012) and microbes become strong sinks of nitrogen (Bardgett et al., 2003).

Brinkman et al. (2010) argues that greenhouse plant–soil feedbacks differ in scale and soil biota composition from natural field experiments due to the inherent differences in geology, biota and climatic conditions including the spectrum and duration of root‐influence on the soil properties. Anaerobic soil conditions caused by high soil moisture or flooding under greenhouse conditions will likely decrease the abundance of the decomposer fungal biomass (Bossio & Scow, 1998). Nevertheless, we proposed a greenhouse experiment using soil analysis, soil sterilization and subsequent addition of inoculum (soil biota originating from the root zone of specific plant species) to reveal the mechanisms responsible for subsequent plant performance (De Long et al., 2019). Confounding biotic and abiotic effects on plant performance in PSFs for the greenhouse experiments were minimized by using independently replicated natural field soils with a long history of *Z. mays* monoculture, pots of similar size, and procedural soil controls (Bardgett & Wardle, 2012; Brinkman et al., 2010).

## 2 MATERIALS AND METHODS

### 2.1 Experimental setup

The greenhouse experiments were conducted at the University of Pretoria (Wheatboard Greenhouse and Labs, S 25°45′21″ E 28°13′51″, South Africa) from 7 March 2018 to 10 December 2018. The topsoil was collected from a monoculture field planted uniformly with *Z. mays* (also called a crop uniformity trial in biometry) at 0–20 cm depth using a soil auger in rows avoiding field borders, unusually eroded areas, wet or dry areas and fertilizer bands. The field layout is a randomized complete block design (RCBD) divided into eight replications separated by 10‐m buffer zone strips in the North–South direction (Eastern Free State, Petrus Steyn in South Africa, GPS: S27°53′45.5″E 28°12′59.8″, Elevation 1705 m). Before using this field soil for the plant–soil feedback experiments, soil analysis showed that most of the selected soil properties of this arenosol grayish sandy soil (FAO, 2005) were ideal to support plant growth (Table 1, Mushonga et al. unpublished).

**TABLE 1:** Biological and physico‐chemical properties of topsoil collected at 0–20 cm depth from a *Zea mays* uniformity trial. The field is located in a grassland biome of the Eastern Free State province of South Africa. *N* = 32 composite samples. SE = standard error

Soil factor	Mean ± *SE*
Clay (%)	8.94 ± 0.18
Silt (%)	9.80 ± 0.24
Sand (%)	81.26 ± 0.26
Density (g/cm^3^)	1.27 ± 0.01
pH(H_2_O)	6.23 ± 0.10
pH(KCI)	4.86 ± 0.07
Phosphate Mehlich III (mg/kg)	48.74 ± 1.46
Phosphate Bray II (mg/kg)	55.41 ± 1.64
Potassium (mg/kg)	87.07 ± 3.25
Cation exchange capacity (cmol(+)/kg)	2.27 ± 0.05
Ammonium nitrogen (mg/l)	0.41 ± 0.003
Nitrate nitrogen (mg/l)	1.54 ± 0.05
Active carbon (mg/kg)	170.93 ± 6.76
Solvita CO₂ respiration (ppm)	4.78 ± 0.23

**FIGURE 1:**
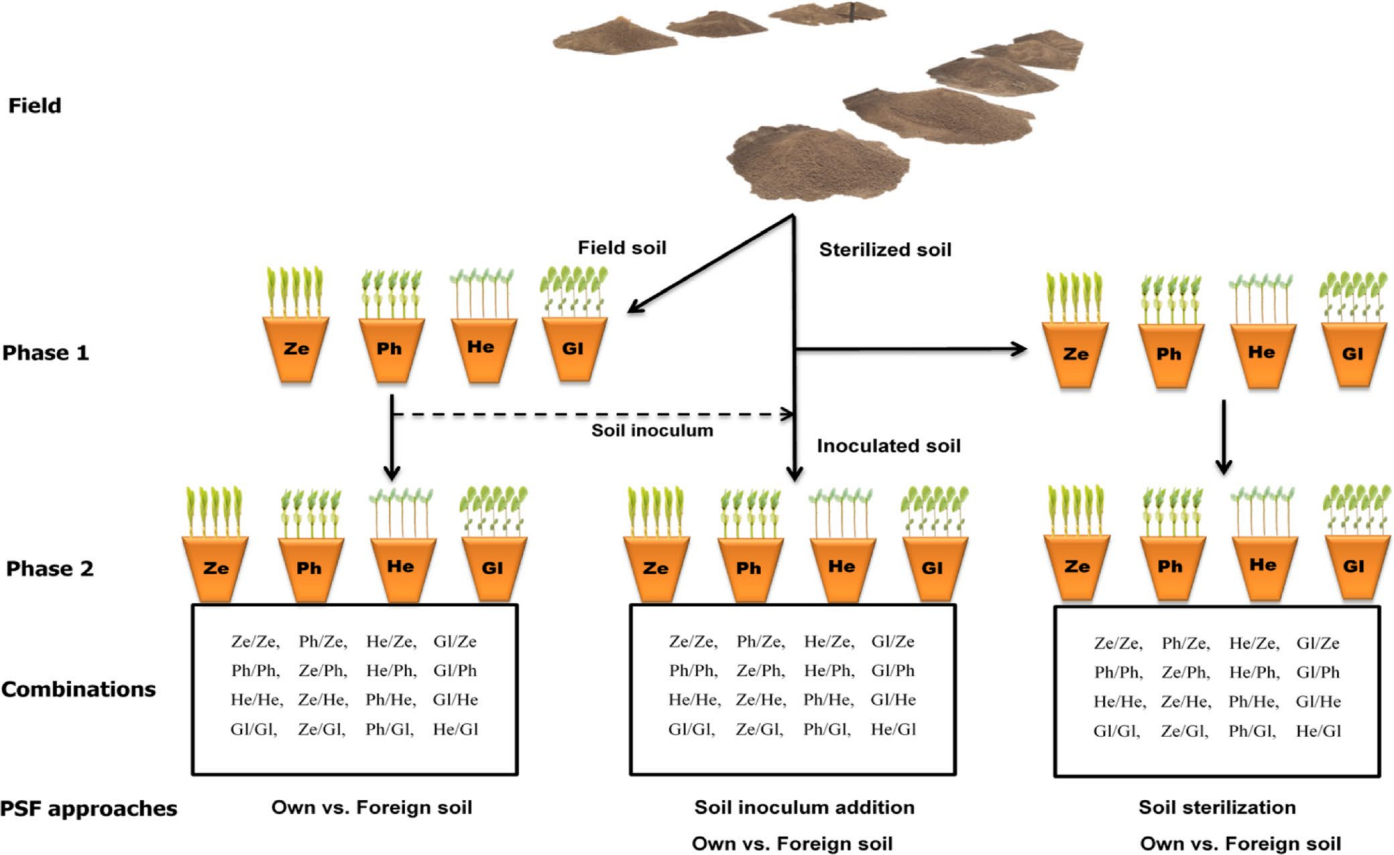
Conceptual diagram (modified from Dias et al., 2014) of the PSF experimental design. In the greenhouse, Phase 1 (conditioning phase) involved cultivating *Z. mays* (Ze), *P. vulgaris* (Ph), *H. annuus* (He), and *G. max* (Gl) in live field soil and sterilized soil. In Phase 2 (feedback phase), the phase 1‐conditioned soils were planted with the same four plant species to give all possible soil combinations of conspecific and heterospecific PSFs. Dashed lines represent soil‐water extract of the inoculum. Drawing courtesy of Mr. Fungai Mushonga

A total of ≈80 kg of this soil (properties in Table 1) was sampled from four treatment plots within each replication following the W‐sampling strategy, air‐dried in a hothouse for a week at 40°C, mixed and sieved using a 5‐mm mesh to homogenize and adjust the soil texture or particle size (Soman et al., 2017; van de Voorde et al., 2011) by removing coarse fragments prior to starting the greenhouse feedback experiments (Figure 1). This reduced confounding spatial effects linked to ecological gradients in soil properties, although other studies have shown that soil structure has no effect in PSF experiments (Bergmann et al., 2016). About 50 kg of soil from each replication was pasteurized in an electrode steam boiler (Model M40, 60 Amps, 40 KW full load at 380 V, 60 kg per hr evaporation from and at 100°C, 550 kPa, Marshal‐Fowler, Randfontein, South Africa) to destroy pathogens, while the remaining 30 kg of soil was left unsterilized (natural field soil). The soil was transferred into pots (size 15 cm diameter and 16 cm depth) and stored until planting. Climate control in the greenhouse environment was adjusted to 25°C maximum air temperature, 7°C minimum air temperature, 93% maximum relative humidity, and 27% minimum relative humidity. This was typical of the Eastern Free State (EFS)‐Petrus Steyn weather according to the Sonop weather station daily data from 23/10/2014 to 02/05/2017. Weather data including air temperature, relative humidity, and solar radiation were collected during the trial period using an Onset HOBO® data logger U12 (MacArthur Blvd).

The plant–soil feedback experiment used the two‐phase approach (Brinkmann et al., 2010); 10‐week conditioning phase and 10‐week feedback phase (Figure 1).

The “unconditioned” control soil representing the natural field/bare soil was potted and stored at the same greenhouse conditions as all pots. These control pots with nothing planted in them throughout the experiment were used to obtain soil inoculum not fed with rhizodeposits. In this way, the impact of the four plant species on soil legacies, which in turn influenced growth of phytometer species, could be differentiated for plant–soil feedbacks.

#### 2.1.1 Conditioning phase (phase 1)

An early medium‐maturity cereal grass (*Z. mays*), two medium‐maturity and indeterminate nitrogen‐fixing leguminous forbs (*P. vulgaris* and *G. max*) and a medium late‐maturity forb (*H. annuus*) were used to study the plant–soil feedbacks due to their good performance in dryland production systems of South Africa. After sowing, plants were watered with reverse osmosis purified water (Pacific AFT 20, 24V, 80W, Thermo Scientific, Niederelbert, Germany) to soil pot capacity on a daily basis in the first week until emergence and thereafter, according to plant need. The inherent challenges of attaining and controlling uniform soil water content in pots (Kramer & Boyer, 1995) was complicated by cultivating the four different plant species (which differ in rates of transpiration) in the same environment. Nevertheless, watering was done according to plant needs at the onset of leaf and plant drooping, and drying of the upper soil in both control and planted pots. The aim was to water plants in a way that minimized drought effects on PSFs (Fry et al., 2018).

The conditioning phase lasted for 10 weeks during which time the four plant species were cultivated in unsterilized (natural) field soil and sterilized field soil with a target plant population of five plants of the same species per pot. In 10 weeks, this planting regime was ideal for conditioning the soil and measuring of plant production metrics for the three functional groups (van de Voorde et al., 2011). Cleaned pots containing 1.3 L soil were set up in a complete randomized block design in a greenhouse (light: 10–12 hr., dark: 12–14 hr.), and watered before planting with 150 ml of purified water. Seeding was done simultaneously at the beginning of each conditioning phase. *Glycine max* seeds were placed with the granular soil inoculant (*Bradyrhizobium japonicum* WB74 strain) in the drilled holes at approximately 5‐cm soil depth. The *G. max* seed was inoculated with 0.4 grams of 1 × 10^9^ colony forming units soil inoculant per gram of seeds at the time of planting using the strain and procedure described and validated for successful nodulation by van der Hoven (2018). The experimental layout (Figure 1) for the first phase had two levels of soil treatments (natural field soil and sterile soil) to grow four plant species (*Z. mays*, *H. annuus*, *P. vulgaris* and *G. max*) replicated 32 times, adding up to 256 pots.

After 10 weeks, plants were harvested and measured for shoot, root and total dry biomass per plant. Soil samples were collected and analyzed for legacies in active carbon (mg/kg), enzymatic activity in FDA hydrolyzed (μg/mg soil), and fungal to bacterial biomass ratio.

#### 2.1.2 Feedback phase (phase 2)

The feedback phase was conducted using two experimental approaches; inoculum addition and sterilization (Figure 1). All the replicates of soil conditioned in phase 1 were air‐dried in pots and stored for 1 month at 12–26°C in situ. This was followed by preparing inoculum from phase 1 conditioned soils for setup of the inoculum addition treatment experiment using a modification of the method described by Bergmann et al. (2016). The soil inoculum represented a microbial filtrate or soil‐water extract/fraction of soil biota originating from the root zone of plant species at the end of the conditioning phase. Soil‐water extract of the inoculum was prepared by mixing composite 2.5 L natural field soil (pooled from 32 replicates) conditioned by *Z. mays*, for example, with 5.0 L deionized water, stirring vigorously for 2 min, allowing to settle for 1.5 min and decanting through a 250 µm sieve. The resulting microbial filtrate was stored at 4°C and used within 2 days to inoculate sterilized field soil for the inoculated soil treatment experiment. These soil‐water extraction steps were repeated for composite soil samples conditioned by the other three species at week 2 and 4 after seedling establishment. Microbial filtrate of 250 ml of donor inoculum was added after seedling establishment at week 2 and 4. *Glycine max* seeds were planted with granular soil inoculant as described in the conditioning phase.

All plant species were allowed to grow for 10 weeks with a target plant population of five plants of the same species per pot. This experiment had 16 plant feedback combinations × 8 replications (*n* = 128 pots) representing the inoculum‐added feedback experiment. Following the inoculum addition experiment, the remaining phase 1 conditioned field soil was replanted to make up 16 feedback combinations representing the non‐sterilized treatment of the sterilization experiment.

Alternatively, the control phase 1 conditioned sterilized field soil was seeded to set up 16 feedback combinations that were replicated eight times (*n* = 128 pots) in the sterilization experiment. Overall, the second phase experimental design had three levels of soil treatments (natural field soil, sterile soil and inoculated soil) each with 16 plant feedback combinations replicated 8 times adding up to 384 pots.

After the 10 weeks of phase 2, the plants were harvested and measured for shoot, root and total dry biomass per plant, while conspecific and heterospecific soil samples were analyzed for legacies in active carbon (mg/kg), enzyme activity in FDA hydrolyzed (μg/mg soil), ammonium nitrogen, nitrate nitrogen, nitrate‐to‐ammonium nitrogen ratio, total nitrogen, and fungal to bacterial biomass ratio. *Helianthus annuus* created a unique legacy of soil properties in the preliminary results at the end of the condition phase. In light of this, further soil fungal and bacterial biomass analysis was done exclusively for selected soils conditioned by *H. annuus* in phase 1.

### 2.2 Plant biomass measurements

Harvesting was done after flowering (for *Z. mays* and *H. annuus*) and at physiological maturity (for *G. max* and *P. vulgaris*) after 10 weeks of plant growth. Biomass metrics of total biomass, above ground (shoot) and below ground (root) biomass were measured and recorded as dry biomass in g per plant. Drying was done at 62°C in a forced‐air oven for 48 hr until constant weight was achieved.

### 2.3 Soil analyses

The active carbon fraction of the organic matter was determined using the permanganate‐oxidizable active carbon method adapted from Weil et al. (2003). For each 2.5 g soil sample, 20 ml of 0.02 M potassium permanganate (prepared using 0.1 M calcium chloride buffer solution) was added and mixed at 120 rpm for 2 min using a rotary shaker. After 8 min of settling the soil suspension, 0.5 ml of liquid fraction was pipetted into 49.5 ml of water to dilute the color of the potassium permanganate. The dilute solutions were measured at 550 nm wavelength using a spectrophotometer (CARY Bio 100 UV‐Vis, Agilent Technologies, USA).

The fluorescein diacetate (FDA) method was used as an indicator of soil enzyme activity, as described by Schnürer and Roswell (1982). Soil samples of 0.5 g were mixed with 20 ml of 60 mM sodium phosphate buffer and 0.2 ml of FDA solution (dissolved in acetone). The mixture was mixed by agitation at 90 rpm for 1 hr in a rotary shaker. The FDA reaction was stopped by aliquoting 2 ml of the soil solutions into sterile 2 ml Eppendorf tubes on ice. The samples were centrifuged at 4,630 g to remove excess debris. The supernatant fractions were measured at 490 nm using a double‐beam spectrophotometer (CARY Bio 100 UV‐Vis, Agilent Technologies, USA).

Phospholipid fatty acid (PLFA) extraction was done as described by Marschner (2007). Frozen field‐moist soil (2 g) was mixed with a citrate buffer, chloroform, methanol, and Bligh and Dyer reagent. Separation of soil and liquid fraction was done by shaking for 2 hr followed by centrifugation at 2,500 g. The supernatant of each sample was evaporated under an N_2_ stream, conditioned with chloroform, and run through an elution chamber. The phospholipids were collected and dried again under an N_2_ stream. An internal standard methylnondecanoate (C19:0) was added along with methanol: toluol, hexane: chloroform, acetic acid, and deionized water. The organic phase was collected and dried under an N_2_ stream. The purified phospholipids already methanolyzed into fatty acid methyl esters, were analyzed on a Varian 430‐GC gas chromatograph (Agilent Technologies, USA) and recorded in nmol/g. In our study, fungal PLFA markers C18:2ω6c, C18:1ω9t, C18:1ω9c, C18:2ω6t and C18:3ω3c, and bacterial PLFA markers C14:0, i‐C15:0, a‐C15:0, C15:0, i‐C16:0, C16:19, C17:0, i‐C17:0 and C18:17 were used (Frostegård and Bääth, 1996; Schwab et al., 2017; Tavi et al., 2013; Willers et al., 2015).

Soil physical, chemical and biological properties were measured using standard procedures at Nvirotek Laboratories (Pty) Ltd (ISO/IEC 17,025 international standard for testing laboratories Accredited Testing Laboratory, certified SANAS and SADCAS assessors, certified AgriLASA Fertilizer Laboratory, Ifafi, South Africa). The soil samples were dried, sieved and analyzed for density prior to preparation of the 1:2 water extract slurry for measuring macro‐ and micronutrients using the Mehlich III extraction method. Total P was determined using the P Bray II method.

### 2.4 Statistics

Data analyses were carried out using R 3.5.2 (R Core Team, 2018), SPSS version 23 (IBM Corp. Released, 2015) and Canoco 5 version 5.11 (ter Braak & Šmilauer, 2018). Time series of climate characteristics were plotted using the “ggplot2” and “tseries” packages in R 3.5.2. Boxplots were drawn in SPSS version 23 for all recorded data to detect outliers in the dataset.

#### 2.4.1 Measure of correlation between phase 1 and phase 2 biomass

Correlation coefficients were calculated to determine the relationships between phase 1 and phase 2 biomass using SPSS version 23. When the data were not normally distributed, the Spearman non‐parametric correlation coefficient was used.

#### 2.4.2 **Test for total biomass and biomass ratio differences between four plant species using the Kruskal**–**Wallis and independent *t*‐test analyses, respectively**

The raw biomass data were sub‐set by soil type and analyzed using a non‐parametric Kruskal–Wallis test to compare biomass per plant across each soil treatment. The root‐to‐shoot and root mass ratios were calculated and used in determining whether there were significant differences between sterilized and field soil within plant groups in the conditioning phase, using the independent *t*‐test analysis in SPSS version 23.

#### 2.4.3 Three‐way (Three factor) analysis of variance to test the main effects on biomass and feedback values

A general linear model with the main effects phytometer species, conditioning species and soil treatment was analyzed to determine the feedback of growing phytometers in natural field soil, sterilized soil and inoculum‐added soil, using a three‐way analysis of variance (ANOVA) in SPSS version 23. Three‐way ANOVA comparisons were computed across phytometers to compare biomass per plant across phytometers after the normality and homogeneity of variance assumptions were met for the original biomass data sub‐set by phytometer plant species.

The feedback calculations were based on biomass variable and feedback variables to *i*th individual observations. The biomass variable was calculated as follows: (O_i_–F_i_) pairwise as described by Brinkman et al. (2010). The feedback variables were calculated as follows; (a) impact of soil sterilization = [(*A*_i_ − *B*_i_)/maximum biomass value of either (*A*_i_ or *B*_i_)], where *A*_i_ is biomass of the phytometer species grown in non‐sterilized soil and *B*_i_ is the biomass of the phytometer species grown in sterilized soil and (b) impact of soil inoculum amendment = [(*A*_i_ − *C*_i_)/maximum biomass value of either (*A*_i_ or *C*_i_)], where *A*_i_ is biomass of the phytometer species grown in soil biota without inoculum and *C*_i_ is the biomass of the phytometer species grown in soil biota with inoculum as described by Brinkman et al. (2010). The strength of PSFs was categorized into non‐significant, significant, highly significant and very highly significant, where *p* > .05, *p* ≤ .05, *p* < .01 and *p* < .001 (IBM Corp. Released, 2015; R Core Team, 2018), respectively.

#### 2.4.4 Paired *t*‐test for differences between “own” and “foreign” biomass data within plant species

Paired differences in biomass per phytometer species group were tested for normality and equal variance assumptions after ignoring significant outliers in the dataset. The biomass data did not violate these assumptions in the paired *t*‐test calculations to determine the strength of plant–soil feedback responses in; (a) “own” relative to “foreign” soil biota and nutrients, (b) “own” relative to “foreign” soil nutrients, and (c) “own” relative to “foreign” soil biota.

#### 2.4.5 **Kruskal**–**Wallis test for differences in soil nitrogen between nine soil origins**

Box plots were used to visualize the descriptive statistics of soil nitrogen, FDA activity and active carbon legacies data using the “Car” package in R 3.5.2 software. Where soil nitrogen data violated the normality assumption, the Kruskal–Wallis test was performed to test for differences in soil nitrogen legacies among nine categories of conspecific/heterospecific soil origin. Where significant differences were detected, pairwise contrasts were calculated to reveal the treatments responsible for the differences.

#### 2.4.6 One‐way ANOVA test for differences in soil active carbon and FDA hydrolyzed between nine soil origins

The ANOVA test for normality was met for both soil active carbon (mg/kg) and FDA hydrolyzed (μg/mg soil) using the Shapiro–Wilk test. The significant ANOVA test results were analyzed using multiple comparisons based on Tukey's honestly significant difference (Tukey HSD) test across soil conditioning and feedback phase's legacies in SPSS version 23.

#### 2.4.7 Ordination technique to rank PLFA data to reveal relationships among selected soil legacies

Multivariate analysis of conspecific/heterospecific soil legacies data for PLFA biomass was visualized using non‐metric multidimensional scaling (NMDS) ordination diagram of the Bray–Curtis dissimilarities, with two computed axes of soil fungal and bacterial biomass across treatments (Canoco 5 version 5.11; ter Braak & Šmilauer, 2018).

## 3 RESULTS

### 3.1 Climate variables between March and September 2018

The mean daily temperature ranges per month for phase 1 was three times that of phase 2 (Figure S1a). However, the daily mean temperatures between seeding and harvesting decreased steadily in phase 1, but increased relatively from the seeding until harvest in phase 2. In Figure S1b, the calculated daily temperature ranges (10.5 to 14.0°C) were strongly influenced by the minima values. In phase 2 (Figure S2a–d), relative humidity ranged from 20% to 70% with a mean of 44%, while the average solar intensity at 10:00 was 7,900 Lux and the dew point mean was 14°C.

### 3.2 Plant performance

The Spearman non‐parametric correlation results indicate that biomass of the first phase and the second phase were not correlated in either sterilized soils (*r_s_* = .095, *n* = 128, *p* > .05) or natural field soils (*r_s_* = .094, *n* = 128, *p* > .05), implying that it is not necessary to include the impact of the first phase nutrient depletion on plant performance in the second (test) phase.

In the conditioning phase, *G. max* total mass was superior to all three other plant species in both field and sterilized soil (Figure S3a–c). Differences in root‐to‐shoot ratios within groups of plant species were not significant, except for *Z. mays* (Table S1; Figure S3d).

### 3.3 Plant performance in response to soil inoculum addition and sterilization

**FIGURE 2:**
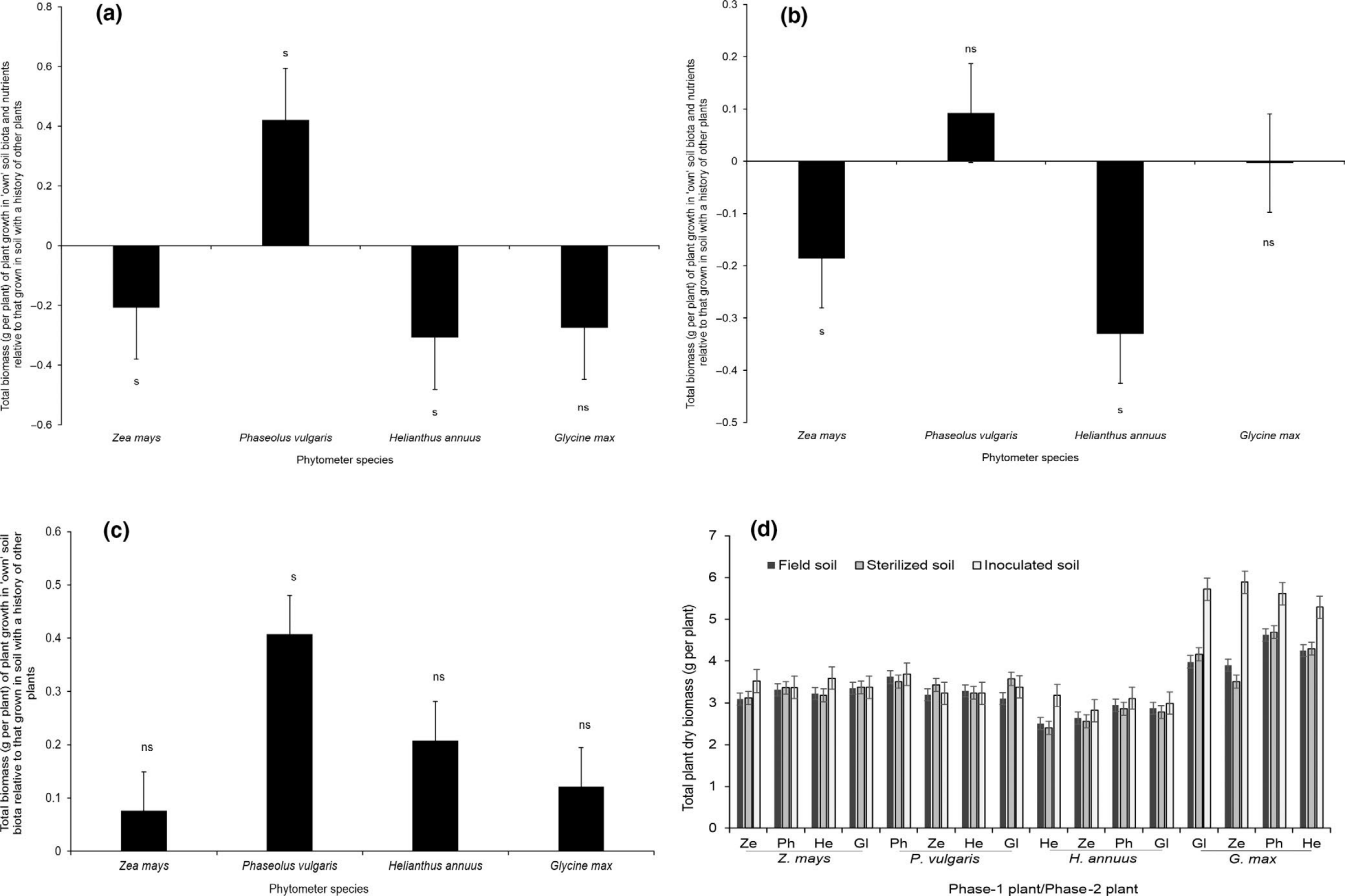
The strength of plant–soil feedback (total biomass) responses of four crop species grown in: (a) “own” relative to “foreign” soil biota and nutrients using natural field soil, (b) “own” relative to “foreign” soil nutrients using sterilized field soil, and (c) “own” relative to “foreign” soil biota using sterilized soil with inoculum added. “s” represents significant differences (*p* ≤ .05) in biomass or a strong PSF value while “ns” represents non‐significant differences (*p* > .05) in biomass or a weak PSF value based on the *paired t‐test* analysis (deviation from zero). (d) The effect of soil treatments on total mass for four phytometer species grown in soils cultivated by their “own” or “foreign” species. *Glycine max* had the highest biomass at the end of the feedback phase. All bars represent the standard error of the mean (*n* = 24 for (a) to (c), *n* = 8 for (d))

The biomass differences among the individual plants of *Z. mays* growing in field soil were found to be significant, while *P. vulgaris*, *H. annuus* and *G. max* were not. The biomass of *Z. mays*, *H. annuus*, *P. vulgaris*, and *G. max*, growing in inoculated soil (Figure 2d) did not differ among each phytometer species. In terms of plant production in all soil treatments, *G. max* produced the highest total dry matter in grams per plant as shown in Figure 2d. *Zea mays* and *P. vulgaris* grew more root than shoot biomass, whereas *H. annuus* and *G. max* produced more above ground than below ground dry matter at the end of the feedback phase.

### 3.4 Comparisons of plant performance across phytometers

A three‐way ANOVA was used to compare the performance of phytometers in the three soil types; natural field soil, sterile soil and inoculated soil (Table S2). The biomass differed between the feedbacks in one or more experimental phytometer groups except *P. vulgaris* (ANOVA test Table S2). Using a three‐way ANOVA and post hoc pairwise tests, the significant differences in *Z. mays* feedbacks were found to emanate from mean differences in monoculture and *H. annuus* conditioned soils in phase 1.

Significant differences in *H. annuus* feedback came from mean differences in monoculture. In *G. max* performance, significant differences in feedback can be explained by mean differences of *Z. mays* conditioned soil and monoculture soil.

### 3.5 Analysis of feedback experiments in monoculture (own) versus crop rotation (foreign) soil

The same plant species were cultivated under similar conditions using different feedback approaches, that is, in sterilized field soil, non‐sterilized soil and soil with inoculum. *Phaseolus vulgaris* consistently showed positive feedbacks in response to both sterilization treatment (Figure 2b,c) and inoculum addition treatment (Figure 2c).

The *paired t‐test* analysis (deviation from 0) of the findings revealed that plant–soil feedbacks varied in direction and strength (Figure 2). *Glycine max* showed a weak negative PSF (*p* > .05) in total biomass (g per plant) (Figure 2a) while *Z. mays* (*p* < .001) and *H. annuus* (*p* < .01) displayed a strong negative PSF. A strong positive PSF was only observed in *P. vulgaris* (*p* < .001; Figure 2a).

The positive PSF performance of *P. vulgaris* in own relative to foreign soil nutrients was very weak, while strong and negative for both *Z. mays* and *H. annuus*. The PSF magnitude of *G. max* was neutral (Figure 2b).

All plant species grew better in their own relative to foreign soil biota (Figure 2c), although with weak positive PSF values for *Z. mays*, *G. max* and *H. annuus*. On the other hand, a strong positive PSF value was observed for *P. vulgaris* (Figure 2c).

Soil treatment (sterilization and inoculum amendment) effects on biomass were significantly different according to three‐way analysis of variance (ANOVA; Table 2).

**TABLE 2:** Three‐way ANOVA (SS type III) table of general linear models for effects on total dry biomass and feedback values associated with soil sterilization and adding soil inoculum. Effect of phytometer species (P; phase 2 *Zea mays* L., *Helianthus annuus* L., *Phaseolus vulgaris* L., *Glycine max* L.), conditioning species (C; phase 1 *Zea mays* L., *Helianthus annuus* L., *Phaseolus vulgaris* L., *Glycine max* L.), soil treatment (S; non‐sterilized soil/soil biota without inoculum/sterilized soil/ soil biota with inoculum), interactions (between; P × C, S × P, S × C, S × P × C). Statistics parameters represented degrees of freedom (*df*), *F*‐statistic and *p* values (*p*). Significant *p* values are <.05

Effect	*df*	Soil sterilization application				Soil inoculum amendment			
		ln‐transformed biomass		Feedback values^a^		ln‐transformed biomass		Feedback values^b^	
		*F*	*P*	*F*	*p*	*F*	*P*	*F*	*p*
Phytometer species (P)	3	97.4	<.001	33.4	<.001	186	<.001	0.834	.478
Conditioning species (C)	3	8.20	<.001	2.07	.108	4.35	.005	0.800	.496
P × C	9	1.77	.076	3.17	.002	0.515	.863	0.815	.604
Soil treatment (S)	1	0.021	.884			55.4	<.001		
S × P	3	0.725	.538			18.8	<.001		
S × C	3	0.538	.657			1.70	.169		
S × P × C	9	0.479	.888			2.47	.011		

^a^ Feedback values represent (*A*_i_ − *B*_i_)/max (*A*_i_, *B*_i_) where A_i_ is biomass of the 2 phytometer species grown in non‐sterilized soil and B_i_ is the biomass of the phytometer species grown in sterilized soil.

^b^ Feedback values represent (*A*_i_ − *C*_i_)/max (*A*_i_, *C*_i_) where A_i_ is biomass of the phytometer species grown in soil biota without inoculum and C_i_ is the biomass of the phytometer species grown in soil biota with inoculum.

For phytometer species, the results of soil sterilization and soil inoculum experimental approaches using log‐transformed total biomass data gave similar results, but very different results in feedback values (Table S2 and Table 2).

### 3.6 Characterization of soil legacies in active carbon and enzyme activity following the conditioning and feedback phase

**FIGURE 3:**
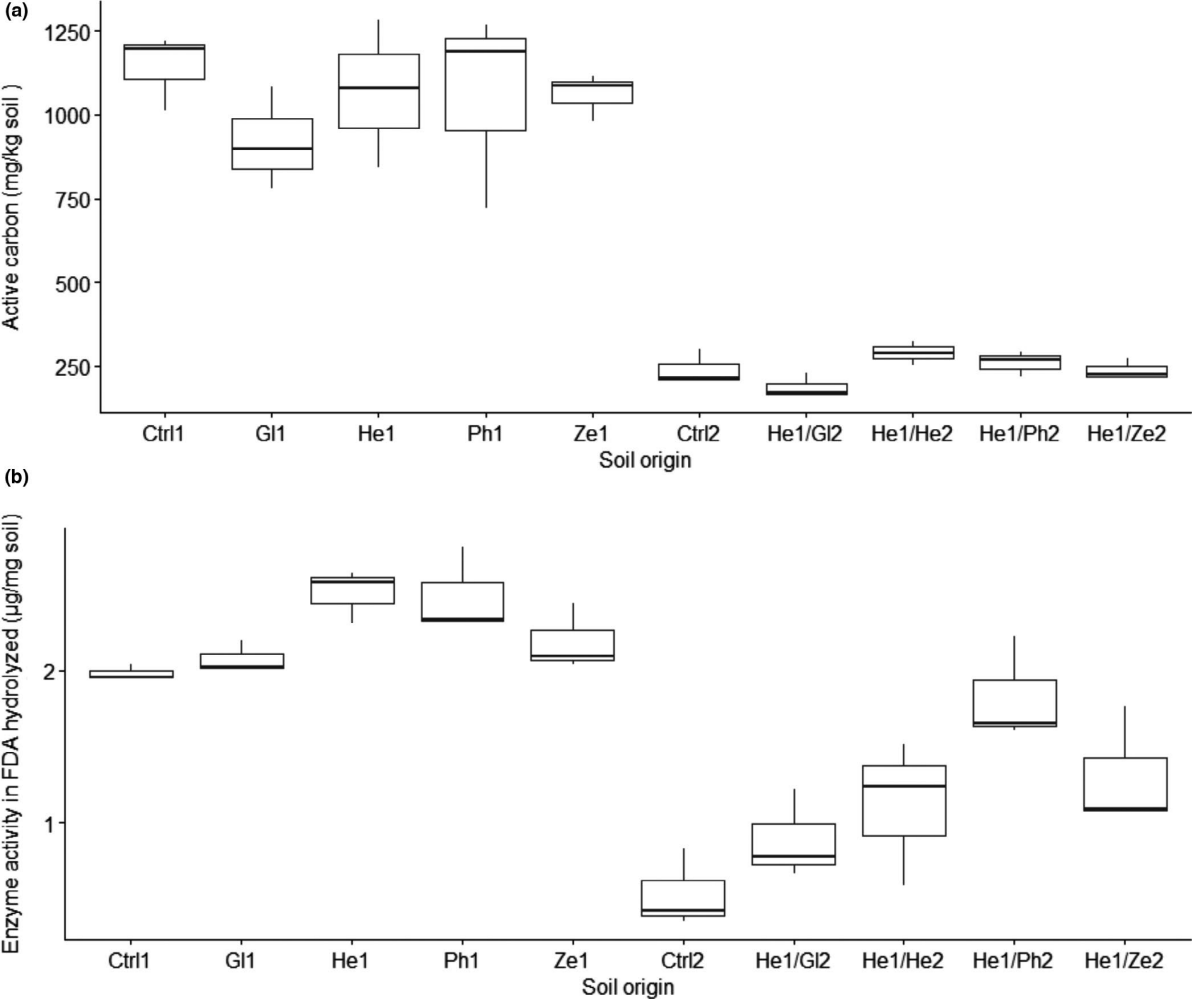
Legacies in soil (a) active carbon (mg/kg) and (b) FDA hydrolyzed (μg/mg soil) at the end of the conditioning (phase 1 = 1) and feedback (phase 2 = 2) phases. After harvest, the active carbon stocks in conditioned soils are lower than the fallow soil (control). The soil enzyme activity (FDA hydrolyzed) legacy is high in all conditioned soils and diminished in the fallow bare soil. Feedback phase legacies were from soils conditioned by *H. annuus* in phase 1. Ctrl – procedural bare control soil, Gl – *G. max*, He – *H. annuus*, Ph – *P. vulgaris*, and Ze – *Z. mays*, He/Ze heterospecific *Z. mays* feedback, He/He conspecific *H. annuus* feedback, He/Ph heterospecific *P. vulgaris* feedback and He/Gl heterospecific *G. max* feedback, horizontal heavy bar = median, box = lower and upper quartiles, whiskers = minimum and maximum values, filled dot = outliers

The average soil legacies in active carbon and enzyme activity following cultivation in phase 1 with all four plant species were 1,026.08 mg/kg and 2.32 FDA hydrolyzed μg/mg soil (Figure 3a,b), respectively. Using the ANOVA test across soil conditioning legacies, the differences in soil active carbon (mg/kg) were not significant (*F_4,10_* = 0.550; *p* > .05) while the enzyme activity in FDA hydrolyzed (μg/mg soil) was found to be significantly different (*F*_4,10_ = 5.10, *p* ≤ .05). The patterns of , shown in Figure 3a,b boxplots.

The *post hoc* multiple comparisons (Tukey's HSD at *p* = .05) for the significant differences in soil FDA hydrolyzed are summarized in Table S3. Feedback soils previously conditioned by *H. annuus* (Table S4) were not significantly different in soil active carbon level but differed significantly in FDA hydrolyzed enzyme activity.

### 3.7 Characterization of soil legacy in nitrogen following the feedback phase

**FIGURE 4:**
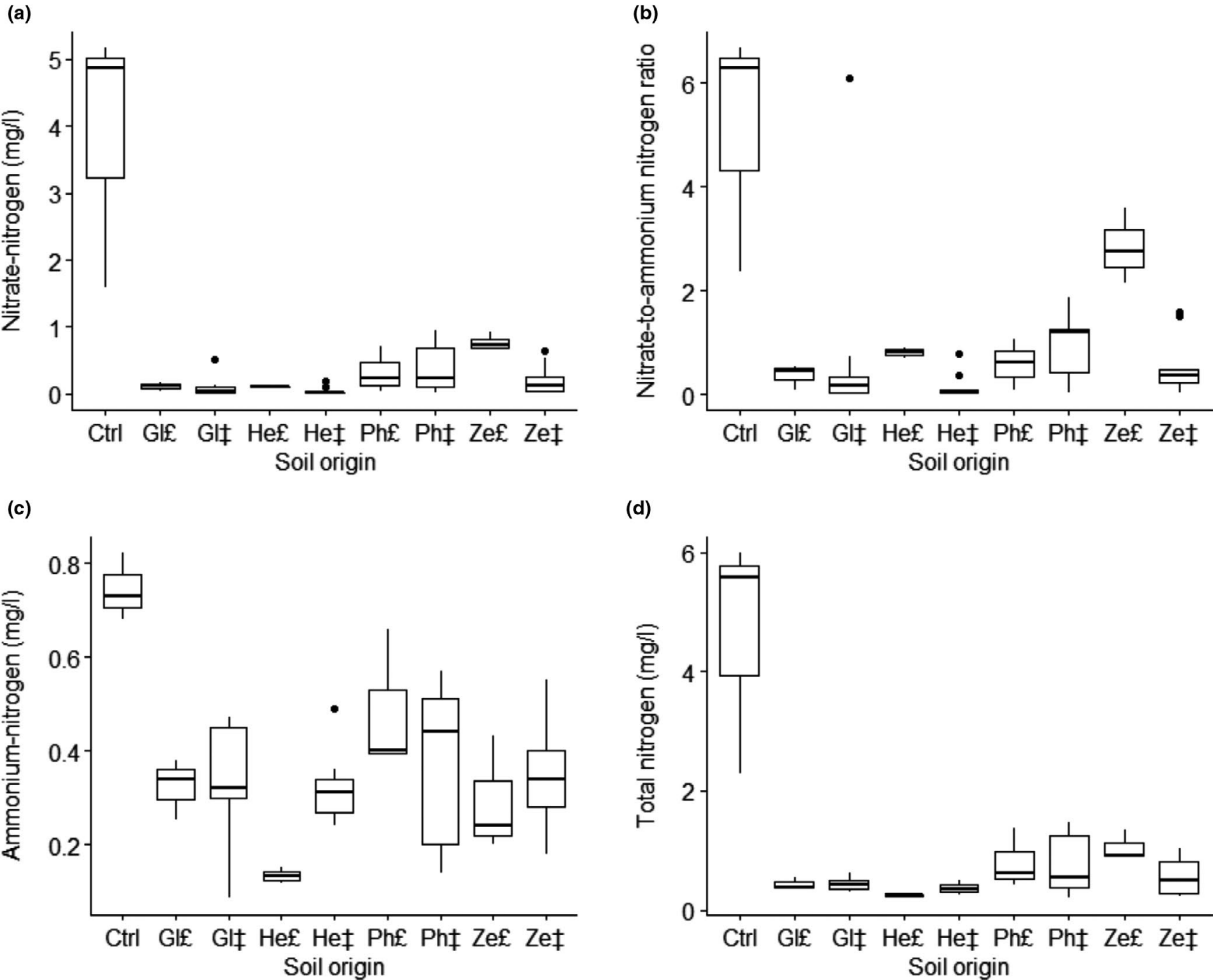
Feedback phase legacy of soil nitrogen in conspecific (₤, *n* = 3) and heterospecific (‡, *n* = 9) plant–soil feedbacks. The soil origin legacies are significantly different in (a) nitrate nitrogen in mg/l (H = 25.4; *df* = 8; *p* < .01), (b) ammonium nitrogen in mg/l (H = 18.9; *df* = 8; *p* < .01), (c) nitrate‐to‐ammonium nitrogen ratio (H = 25.4; *df* = 8; *p* < .01), (d) total nitrogen in mg/l (H = 24.0; *df* = 8; *p* < .01). Horizontal heavy bar = median, box = lower and upper quartiles, whiskers = minimum and maximum values, filled dot = outliers

Having calculated the averages in soil nitrogen legacies after the second phase, total soil N in control soil increased to 4.62 mg/L (Figure 4d) from the initial 1.95 mg/L total soil N in field‐sampled soil (Table 1). Soil total nitrogen legacies were on average highest in control natural field soil (4.62 mg/L), followed by *Z. mays* conspecific soil (1.06 mg/L), *P. vulgaris* conspecific (0.81 mg/L) and heterospecific soils (0.78 mg/L), as shown by boxplots in Figure 4d. The Linnaeus grass (*Z. mays*; Ze) and legumes (*P. vulgaris*; Ph and *G. max*; Gl) had a relatively high total nitrogen legacy compared to the forb (*H. annuus*; He). This suggests that plant growth in this experiment was not limited by soil nitrogen availability during the feedback phase experiment.

In the Kruskal–Wallis test results, the level of soil nitrogen was not the same (*p* ≤ .05, Table S5) among the nine categories of soil origin. The pairwise contrasts (Table S6) show that these significant results in soil nitrate nitrogen feedbacks were between the *H. annuus* heterospecific soil and conspecific *Z. mays* soil pair, *H. annuus* and control natural field soil pair, including the pair of heterospecific *G. max* soil and control natural field soil. There was a significant difference in soil ammonium nitrogen legacy (Figure 4c; Table S6) between *H. annuus* conspecific soil and natural field soil.

### 3.8 Soil microbial biomass legacy following the conditioning and feedback phase

The total PLFA biomass patterns and very low ratios for bacteria indicate that all soils were dominated by Gram‐negative (GN) bacteria biomass compared to Gram‐positive (GP) bacteria (Table S7). As expected, in the first phase, fungal biomass dominated over bacteria in all the soil histories except control soil (Table S7). However, there was a shift towards bacterial dominance at the end of phase 2 for all heterospecific soils conditioned by *H. annuus* in phase 1 (Table S7). *Zea mays* supported more biomass of viable bacteria and fungi than the three other plant species.

Surprisingly, some fungal PLFA biomarker legacies for C18:3ω3c were reduced in all treatments, except conspecific *H. annuus* (He/He), while others like C18:1ω9t disappeared completely in all treatments of phase 2 soils previously conditioned by *H. annuus* (Table S8). In bacterial PLFA biomarker legacies, a‐C15:0 and i‐C15:0 disappeared in all except heterospecific *Z. mays* (He/Ze) soil; markers C15:0, i‐C16:0 and C17:0 disappeared in all the conspecific and heterospecific treatment soils conditioned by *H. annuus*; biomarker i‐C17:0 was absent in both phases and biomarker C18:17 appeared in the second phase although it was absent in phase 1 in all soil treatments.

Fungal and bacterial PLFA biomarkers from soils conditioned in phase 1, and conspecific and heterospecific feedback soils conditioned by *H. annuus* in phase 1 revealed that C16:19 biomarker was two‐fold higher in bare control soil, while C17:0 was absent at the end of the conditioning phase (Table [Link], [Link]). Fungal biomarker C18:3ω3c was unique to *H. annuus* conspecific soils while the marker C18:2ω6c was 10 times higher in conspecific *H. annuus* soil than the remaining heterospecific soils. Bacterial markers i‐C15:0 and a‐C15:0 were unique to heterospecific *Z. mays* soils. The bacterial C14:0 biomarker was absent in both conspecific *H. annuus* soil and heterospecific *P. vulgaris* soils (Table [Link], [Link]).

**FIGURE 5:**
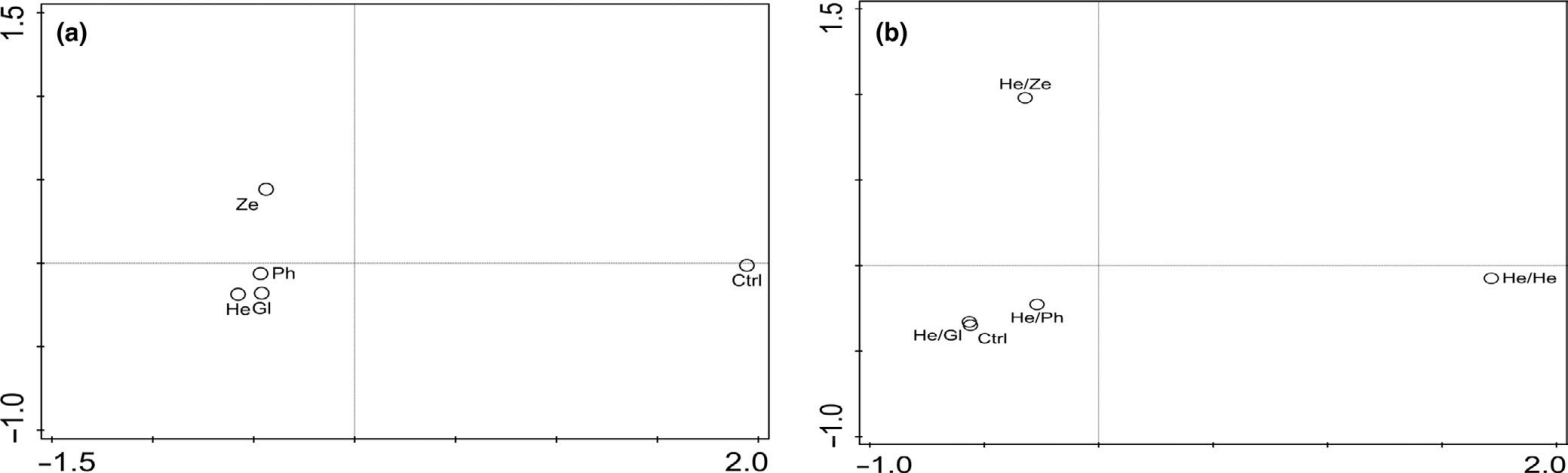
Non‐metric multidimensional scaling (NMDS) ordination diagram of the *Bray*–*Curtis* dissimilarities with two computed axes of microbial biomass of fungi and bacteria PLFAs in soils after the conditioning phase (a) and the conspecific *H. annuus* and heterospecific soils after the feedback phase (b). Ctrl – procedural bare control soil, Gl – *G. max*, He – *H. annuus*, Ph – *P. vulgaris*, and Ze – *Z. mays*, He/Ze heterospecific *Z. mays* feedback, He/He conspecific *H. annuus* feedback, He/Ph heterospecific *P. vulgaris* feedback and He/Gl heterospecific *G. max* feedback

Similarities among phase 1 conditioned soils (Figure 5a) and among phase 2 soils conditioned by *H. annuus* (Figure 5b) were based on soil microbial biomass legacies. The unlabeled first NMDS axes are strongly correlated with fungal and bacterial biomass legacies in soil (Figure 5), with highest dissimilarities between the bare control soil and soils conditioned by the four plant species (Figure 5a; stress: 0.0001, *k* = 2).

However, microbial biomass legacies in soils conditioned by the plant species were ordinated closer to each other, suggesting that they were less dissimilar. After the feedback phase, the microbial biomass legacies in conspecific *H. annuus* soil were dissimilar from other heterospecific feedbacks. Moreover, the heterospecific *Z. mays* soil was dissimilar from the cluster of heterospecific *G. max*, *P. vulgaris* and bare control soil (Figure 5b; stress: 0.0000, *k* = 2).

## 4 DISCUSSION

### 4.1 Plant performance

In this greenhouse study, live and sterilized field soil were conditioned under mean daily temperature of 28°C and soil moisture regime which were optimum for plant growth, preexisting soil pathogens and mutualists (in live soils), including the symbiont *B. japonicum* (for *G. max*). However, these favorable glasshouse conditions are known to trigger transition of arbuscular mycorrhizal associations from mutualistic to neutral and eventually to parasitic trophic relationships with plant partners (*Klironomos*, 2003). The superior total dry biomass of *G. max* is worth considering for soils requiring increased litter inputs for organic matter improvements.

### 4.2 Feedback experiments

In this study, the high values in biomass feedbacks in soil with added inoculum compared to those in sterilized soils indicate that microbiota support all crop species at larger magnitudes in live soils, confirming that microbes play a role in PSFs (Figure 2). Furthermore, these observed positive effects in own soil microbiota suggest that mutualists had a greater impact on plant performance than pathogens. The biological activities of the soil organisms in the rhizosphere correlate to a decrease or increase in plant performance (De Long et al., 2019). However, the overall soil biotic effects in plant–soil feedback depend on the net‐activity of pathogens, antagonists, mutualists, and decomposers (van der Putten et al., 2013). Destroying these groups of soil biota, for instance through soil sterilization, minimizes the impact of negative plant–soil feedback on plant performance (Cesarano et al., 2017).

*Glycine max* unexpectedly showed negative, neutral and positive feedbacks despite artificial inoculation with symbiotic *B. japonicum* in field, sterilized and inoculum‐added soils, respectively (Figure 2). However, other studies have shown that leguminous nitrogen‐fixing species also develop negative plant–soil feedbacks under monoculture through self‐inhibitory effects (Cesarano et al., 2017).

For *P. vulgaris*, it is clear from parallel experimental approaches, soil sterilization, addition of soil inoculum, and soil conditioning by “own” versus. “foreign” plant species, that the consistent positive soil feedback results were strongly associated with its own beneficial soil microbiota which in turn promoted plant growth (Baxendale et al., 2014). In other words, the conditioning phase legacy of mutualists and decomposers was more significant than pathogens (Brinkman et al., 2010). On the other hand, the rest of the phytometer species were negatively affected by the combination of conspecific soil microbiota and nutrients (Figure 2a). This seems to indicate that *Z. mays*, *H. annuus* and *G. max* perform best in soils cultivated in plant rotations by avoiding injuries due to species‐specific pathogens or allelopathy common in monoculture (Bardgett & Wardle, 2012). We propose that this negative PSF was possibly due to host‐specific pathogens and host‐specific shifts in microbial biomass and composition (Bardgett, 2009).

In this study, the inoculum addition approach produced feedback results that were more positive in all plant species than in the sterilization approach (Figure 2b,c), suggesting that the effects of mutualists/beneficials are less density‐dependent than are soil pathogen effects (Brinkman et al., 2010). The inoculum addition approach can isolate microbial effects from physico‐chemical effects, although the resulting soil conditions will favor fast‐growing microbes and plant species (Forero et al., 2019).

*Zea mays* and *P. vulgaris* had a consistently higher root mass than *H. annuus* and *G. max* in all soil treatments at the end of the feedback phase. This high root‐trait response is associated with adaptation to low nutrient supply and soil carbon budgets (Lambers et al., 2008). Other studies show that the high specific root length (SRL) and finer roots of grasses increase their susceptibility to pathogens and the ability to acquire nutrients from soil (Bergmann et al., 2016) in conspecific soils, while promoting positive plant–soil feedbacks in heterospecific soils (Kos et al., 2015). Monocot grasses have a more fibrous root system than most eudicots, which have a less dense but deeper root system (Fageria, 2013). Deep roots of forb species in conspecific soils reduce nutrient availability and increase species‐specific pathogenic fungi responsible for negative plant–soil feedbacks (Kos et al., 2015). Results showed that there was no correlation between phase 1 and phase 2 biomass responses, indicating that soil nutrient depletion in the conditioning phase did not go on to affect the phytometer species in the feedback phase (Kardol et al., 2006). The overall feedback effects suggest mutualistic interactions between crops and soil organisms in combination with the abundance of these specific soil biota (Brinkman et al., 2010).

### 4.3 Soil legacies

*Zea mays* cultivated soils had similar high soil active carbon stocks to *H. annuus* and *P. vulgaris*. C4 plants such as *Z. mays* have a high turnover of carbon and their root exudates are rich in amino acids, sugars and organic acids (Baudoin et al., 2003). These simple soluble substrates provide nourishment for pioneer saprophytic fungi (Deacon, 2006).

From our observations, the low active carbon stocks in soils cultivated with *G. max* at the end of the conditioning phase (Figure 3a) may predispose microbes to mineralization of the dissolved organic N and excretion of ammonium ions. In this way, microbes meet their energy needs for growth and increase nitrogen legacies in the soil (Bardgett, 2009).

Soil legacies of microbial communities supported by plant roots include the dominating and fast‐growing Gram‐negative bacteria (de Boer et al., 2005) which possibly survive on relatively labile active carbon (Fanin et al., 2019). The relative fungal to bacterial biomass ratio in conspecific *H. annuus* soils compared to heterospecific soils of *Z. mays*, *G. max* and *P. vulgaris* showed that this forb species had either unique or abundant soil fungal and bacterial composition. In addition, conspecific *H. annuus* soils had the highest legacy of soil microbial enzyme activity in the first phase, which in turn possibly created high legacy of active carbon stocks at the end of phase 2. Increasing soil fungal to bacterial ratios are linked to litter decomposition and increasing carbon storage (Malik et al., 2016). The decomposition process is also favored over plant biomass growth by abiotic factors such as high moisture and mean daily temperatures (FERTASA, 2016).

Disappearance of some fungal and bacterial PLFA biomarkers in soils at the end of the second phase indicates that they were sensitive to a factor(s) not identified in this study. Moreover, we did not record changes in soil pH or the accumulation of allelochemicals. However, bacterivorous nematodes of the genera *Acrobeloides*, *Cephalobus* and *Acrobeles* were the most prevalent in these soils (Marais et al. data not published) and probably contributed in part to the disappearance and reduction of bacterial biomarkers. Zhang et al. (2015) also reported that *Z. mays*‐*G. max* rotations supported dominance of *Acrobeloides* in the soil. Generally, all treatments had a higher soil fungal and bacterial biomass legacy than bare control soil, indicating that the plant‐derived substrates from rhizodeposition supported an increase in soil microbial composition and biomass (Baudoin et al., 2001).

Our results also detected a 10‐fold increase in the fungal lipid biomarker linoleic acid (C18:2ω6c, methyl cis‐9–12‐octadecadienoate) in soil for *H. annuus* monoculture, compared to the remaining heterospecific soils. This is a biomarker of saprophytic fungi (Frostegrard and Bääth, 1996; Schwab et al., 2017; Tavi et al., 2013; Willers et al., 2015) whose activities are to mineralize nutrients necessary for plant growth, which in turn support soil biological communities (Bardgett & Wardle, 2012). *Helianthus annuus* monoculture also established a unique linolenic acid (C18:3ω3c, methyl cis‐9–12–15‐octadecatrienoate) soil fungal biomarker (Schwab et al., 2017). The relative increase in common and reliable fungal markers in *H. annuus* conspecific soils compared with heterospecific soils may explain the observed high feedbacks in soil enzyme activity and active carbon stocks. These soil legacies are essential ecosystem services in building organic matter for plant nutrition, especially in dryland sandy soils of South Africa, where farmers are faced with the problem of naturally low soil organic matter varying between 0.5% and 3% (FERTASA, 2016). The PLFA data from this greenhouse study were unreplicated, as such, extra caution must be taken to avoid misinterpreting results. However, these PLFA findings confirmed patterns which were observed in the field during the growing season in February 2018 from *Z. mays* monoculture and crop rotation systems replicated eight times (Steyn et al., 2019).

It is possible that the root traits of *H. annuus* enabled the crop to efficiently extract mineral nutrients, leading to the observed poor soil nitrogen legacies and an increase in fungal to bacterial biomass ratio. Soil carbon legacies of a grass monoculture favor bacteria, actinomycetes and fungal biomass growth more than a legume and a forb (Ladygina & Hedlund, 2010). This is in agreement with our results whereby *Z. mays* grass had the highest total PLFAs relative to the other three species. The decline in soil active carbon and PLFA legacies after the second phase suggests that remaining microbial groups overcame possible selection pressure due to competition for diminishing labile carbon stocks in the decomposition sequence during microbial succession (Deacon, 2006).

In the conditioning phase, the higher FDA hydrolysis in soil legacies for *P. vulgaris* and *H. annuus* than the other two species indicate that they are capable of cultivating suppressiveness to pathogens in soil associated with relatively high enzyme activity (Janvier et al., 2007). The soil legacies in nitrate nitrogen and ratio of nitrate nitrogen‐to‐ammonium nitrogen were significantly higher in conspecific *Z. mays* soil than *H. annuus* heterospecific soil (Figure 4), suggesting that monoculture of *Z. mays* is better at increasing nitrate stocks, which in turn can alkalinize the rhizosphere better than *H. annuus* (Ryan et al., 2009). This indicates that *Z. mays* monoculture removes less nitrogen from the soil than other plants, hence, rotating *Z. mays* with nitrogen‐fixing legume species, specifically *G. max,* will be a good option for growers as a way of reducing costs on nitrogen spending in fertilizer programmes (FERTASA, 2016).

The microbial biomass legacies in *H. annuus* soil under monoculture were different from other heterospecific soil feedbacks. The heterospecific *Z. mays* soil was also dissimilar from heterospecific *G. max*, *P. vulgaris* and bare control soils (Figure 5). It is still difficult to generalize that soil fungal to bacteria biomass ratios can act as indicators of soil health in PSFs since thresholds are not yet clear for their transitions in soils (Bardgett & McAlister, 1999). Additional information is required on the taxonomy and microbial activities of these true markers for fungi in *H. annuus* monoculture using culture‐dependent and –independent techniques in soil microbial ecology. In summary, *H. annuus* seems to cultivate a significantly high biomass of viable saprophytic fungal markers in addition to an enzymatically active soil legacy that may be crucial in building soil organic matter under monoculture. However, there is evidence that positive PSFs are associated with the influence of soil mutualists (Brinkman et al., 2010).

## 5 CONCLUSIONS

These greenhouse‐measured PSFs demonstrate that plant species induce changes in legacies of soil fungi, bacteria, active carbon and enzyme activity that may in turn influence overall soil health and plant health in soil–plant systems. Following the sterilization and inoculum addition approaches, the direction and strength of PSF results were similar for *P. vulgaris*, but different for *Z. mays*, *H. annuus* and *G. max*. However, the consistent positive PSF results in “own” compared to “other” soils under the inoculum addition approach suggest that soil microbial effects were largely due to mutualist effects, rather than species‐specific pathogens. In sterilized soils, only *P. vulgaris* grows well in monoculture through positive PSFs, while the other remaining three species grow best in crop rotations through neutral to negative PSFs. Our results show that *H. annuus* was superior to other crop species in cultivating relatively high active carbon stocks and an enzymatically active soil for the next crop. In this context, increasing the frequency of *H. annuus* in rotation sequences can raise fungal relative to bacterial biomass in soils, while *Z. mays* can increase total microbial biomass. However, more studies using Koch's postulates and plant tissue analysis are needed to evaluate whether the effects of elevated fungal to bacterial biomass ratios in soils promote or depress plant performance and nutrition under different environments. In addition, in vitro plate assays and soil bioassays are necessary to test soil legacies for suppressing soil‐borne pathogens. In spite of the need to confirm these greenhouse findings under field conditions, we make three suggestions to growers, especially in dryland cropping systems. Firstly, *H. annuus* can recruit and feed decomposer fungi, raise soil enzyme activity and build active carbon stocks. Secondly, *Z. mays* will likely enrich the soil with a balanced microbial community and diversity (Baudoin et al., 2001) and minimize removal of soil nitrogen from sandy soils with low organic matter. Thirdly, *G. max* may increase the quantity of litter inputs at the end of the harvest period.

## CONFLICT OF INTEREST

The authors have no conflict of interest to declare.

## Supporting information

Fig S1

Fig S2

Fig S3

Table S1

Table S2

Table S3

Table S4

Table S5

Table S6

Table S7

Table S8
