# Supplementary figures and images for: Plant–soil feedback responses of four dryland crop species under greenhouse conditions
Source: Plant Environ Interact. 2020 Dec 7;1(3):181–95. doi: 10.1002/pei3.10035 (PMC10168064; doi:10.1002/pei3.10035)

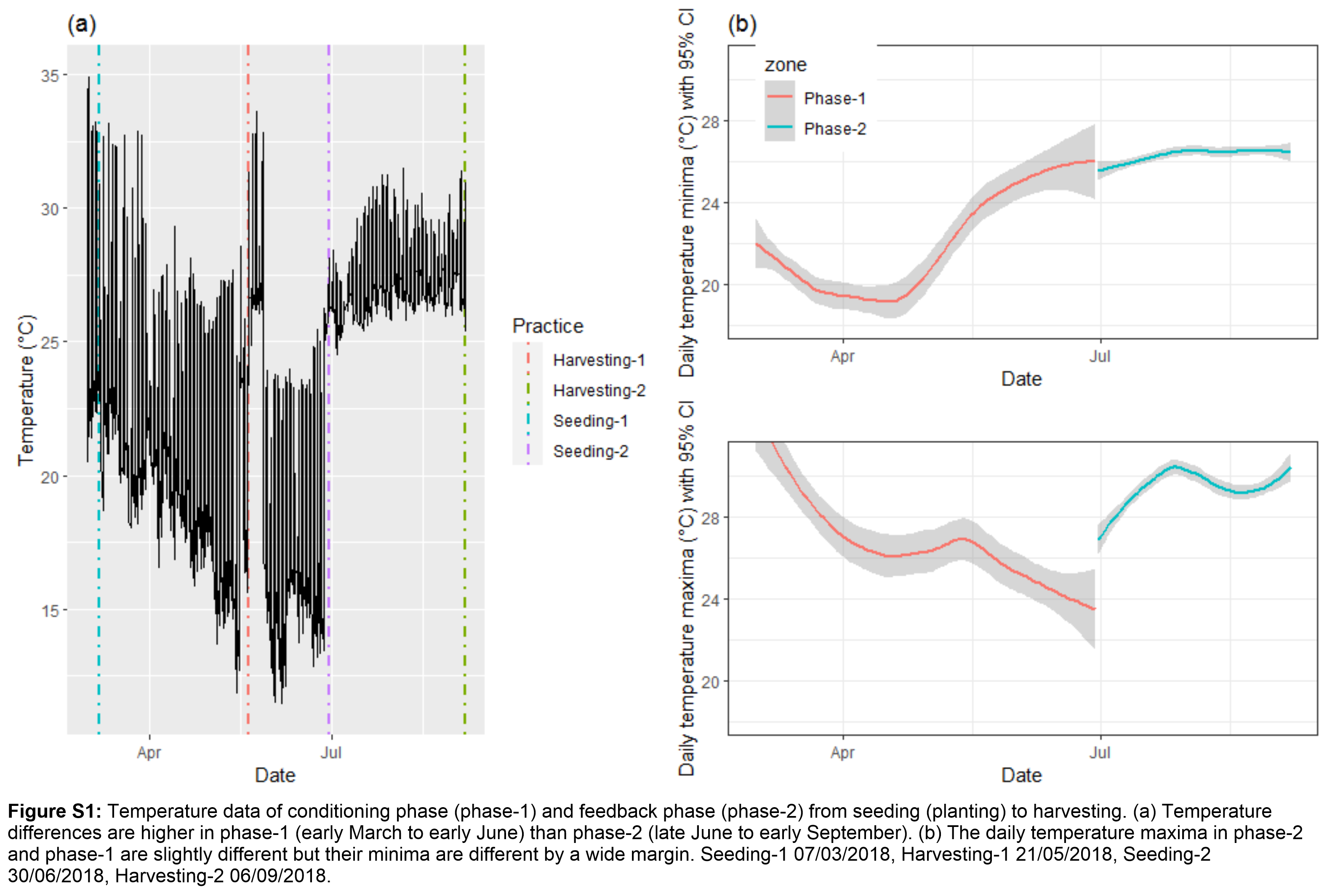

Supplement: Supplementary file 1 — Fig S1 [file PEI3-1-181-s008.tif]

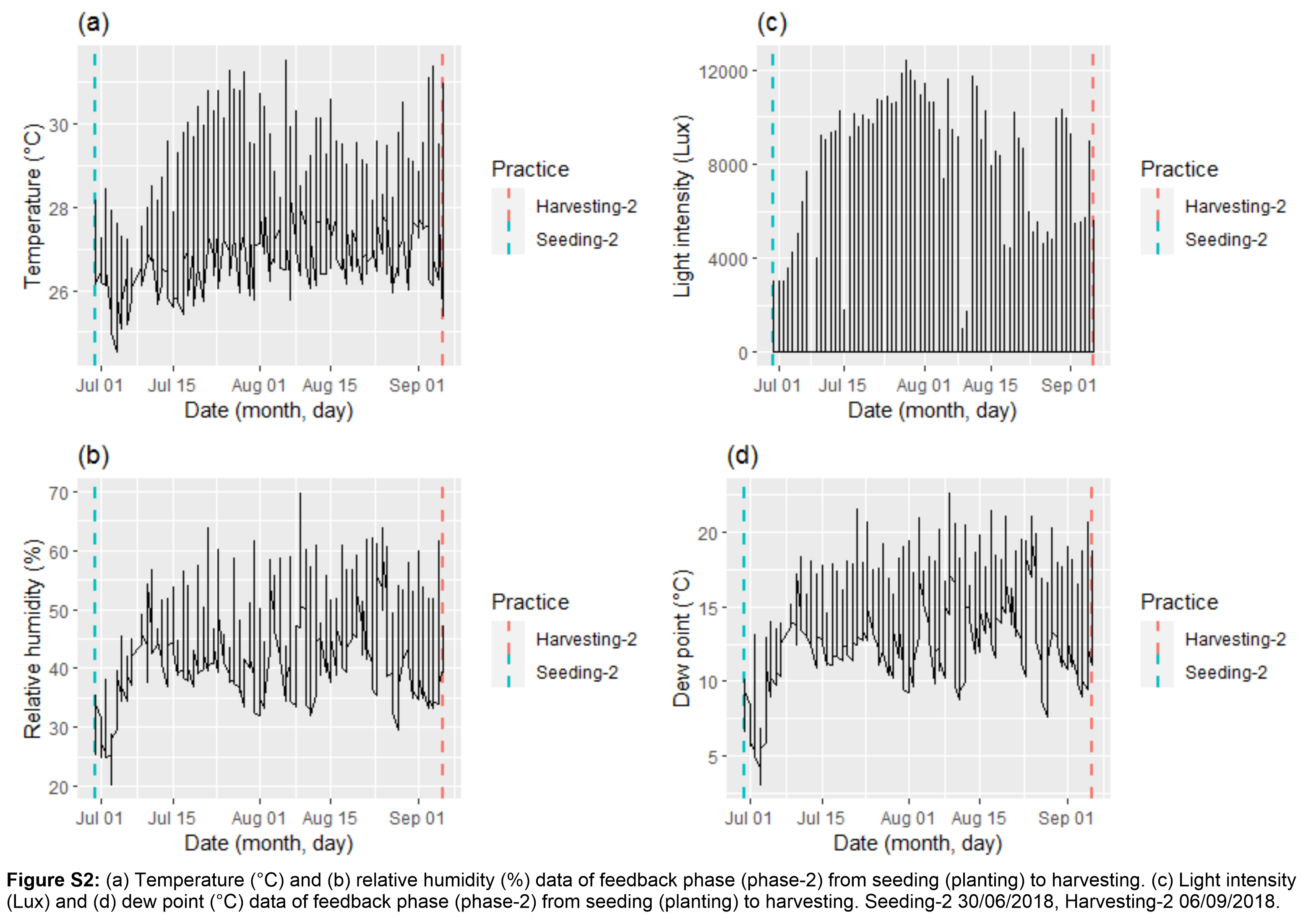

Supplement: Supplementary file 2 — Fig S2 [file PEI3-1-181-s007.tif]

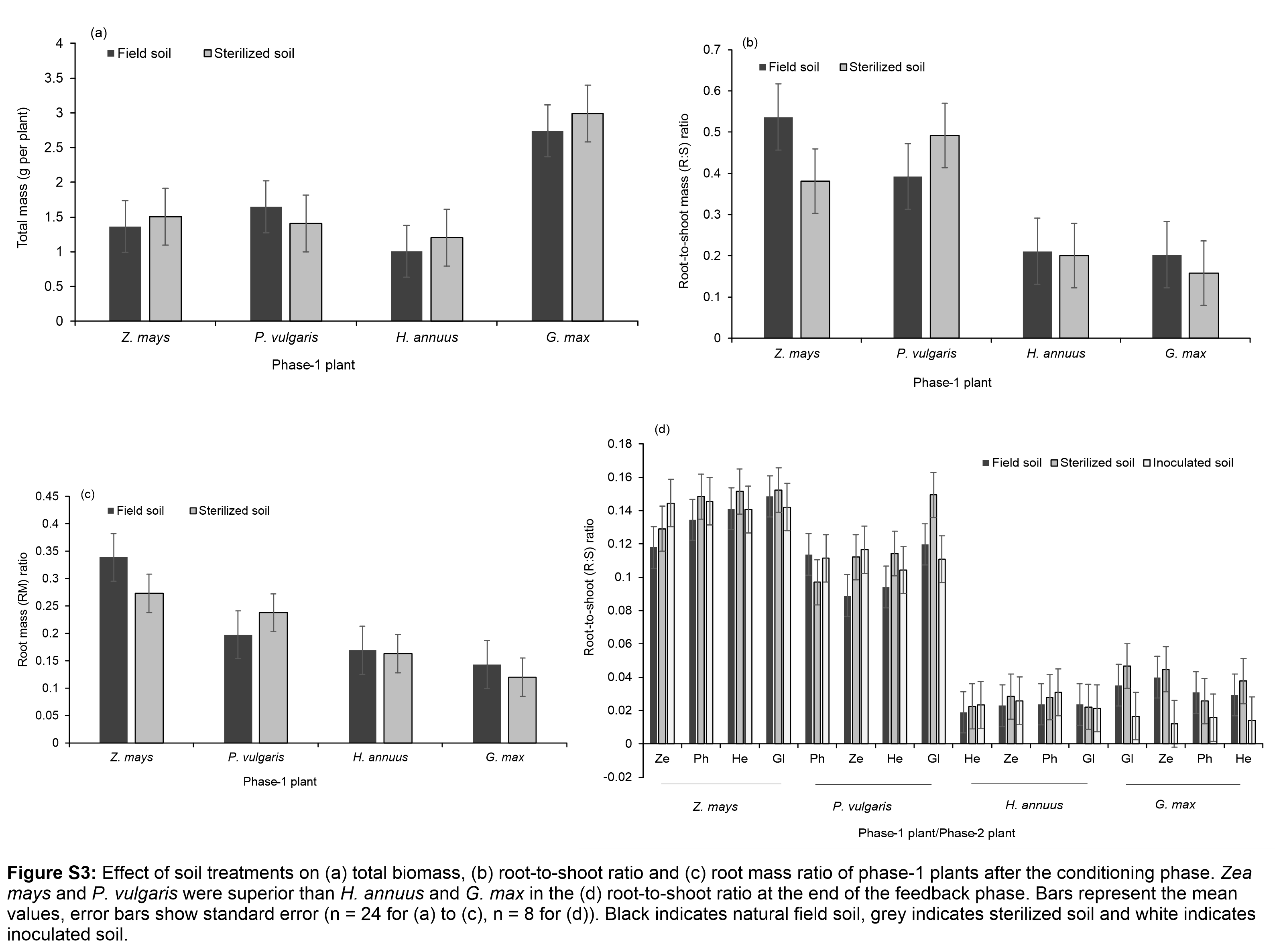

Supplement: Supplementary file 3 — Fig S3 [file PEI3-1-181-s006.tif]
