# Supplementary material for: Plant–soil feedback responses of four dryland crop species under greenhouse conditions
Source: Plant Environ Interact. 2020 Dec 7;1(3):181–95. doi: 10.1002/pei3.10035 (PMC10168064; doi:10.1002/pei3.10035)
Supplement: Supplementary file 4 — Table S1 [file PEI3-1-181-s010.docx]

| Plant species | Root to shoot ratio | | Root mass ratio | |
| --- | --- | --- | --- | --- |
|  | *t_62_* | p | *t_62_* | p |
| *G. max* | 1.544 | 0.128 | 1.499 | 0.139 |
| *H. annuus* | 0.488 | 0.627 | 0.425 | 0.672 |
| *P. vulgaris* | -1.498 | 0.139 | -1.562 | 0.123 |
| *Z. mays* | 3.892 | <0.001 | 4.207 | <0.001 |

**Table S1:** Independent *t*-test analysis of the effect of soil sterilization on plant performance (total biomass, root-to-shoot ratio and root mass ratio) of *G. max*, *H. annuus*, *P. vulgaris* and *Z. mays* in the conditioning phase. Significant *p* values are < 0.05.
