# Supplementary material for: Plant–soil feedback responses of four dryland crop species under greenhouse conditions
Source: Plant Environ Interact. 2020 Dec 7;1(3):181–95. doi: 10.1002/pei3.10035 (PMC10168064; doi:10.1002/pei3.10035)
Supplement: Supplementary file 5 — Table S2 [file PEI3-1-181-s001.docx]

| Experimental group | | Total biomass | |
| --- | --- | --- | --- |
| Phytometer species | **Conditioning species** | ***F*** | **p** |
| *Zea mays* | *Zea mays* | 7.24 | 0.004 |
| *Zea mays* | *Phaseolus vulgaris* | 0.202 | 0.819 |
| *Zea mays* | *Helianthus annuus* | 22.4 | <0.001 |
| *Zea mays* | *Glycine max* | 0.047 | 0.954 |
| *Phaseolus vulgaris* | *Zea mays* | 0.927 | 0.411 |
| *Phaseolus vulgaris* | *Helianthus annuus* | 0.057 | 0.945 |
| *Phaseolus vulgaris* | *Glycine max* | 3.07 | 0.068 |
| *Phaseolus vulgaris* | *Phaseolus vulgaris* | 0.194 | 0.825 |
| *Helianthus annuus* | *Zea mays* | 1.58 | 0.229 |
| *Helianthus annuus* | *Helianthus annuus* | 9.79 | 0.001 |
| *Helianthus annuus* | *Glycine max* | 0.480 | 0.626 |
| *Helianthus annuus* | *Phaseolus vulgaris* | 0.848 | 0.442 |
| *Glycine max* | *Zea mays* | 29.9 | <0.001 |
| *Glycine max* | *Helianthus annuus* | 3.31 | 0.056 |
| *Glycine max* | *Glycine max* | 12.5 | <0.001 |
| *Glycine max* | *Phaseolus vulgaris* | 2.99 | 0.072 |

**Table S2:** ANOVA test results of plant-soil feedbacks among three soil types (field, n = 8; sterilized, n= 8; inoculated, n = 8) within groups of phytometer species using original total biomass data. Significant *p* values are < 0.05.
