# Supplementary material for: Plant–soil feedback responses of four dryland crop species under greenhouse conditions
Source: Plant Environ Interact. 2020 Dec 7;1(3):181–95. doi: 10.1002/pei3.10035 (PMC10168064; doi:10.1002/pei3.10035)
Supplement: Supplementary file 6 — Table S3 [file PEI3-1-181-s002.docx]

| Soil origin | Mean difference | Confidence interval | *P* value |
| --- | --- | --- | --- |
| He – Ctrl | 0.530 | [0.033, 1.028] | 0.035 |
| He – Gl | 0.437 | [-0.061, 0.934] | 0.093 |
| He – Ph | 0.020 | [-0.477, 0.517] | 1.000 |
| He – Ze | 0.318 | [-0.180, 0.815] | 0.290 |
| Ph – Ctrl | 0.510 | [0.013, 1.007] | 0.044 |
| Ph – Gl | 0.416 | [-0.081, 0.913] | 0.114 |
| Ph – Ze | 0.297 | [-0.200, 0.794] | 0.345 |
| Ze – Ctrl | 0.213 | [-0.284, 0.710] | 0.636 |
| Ze – Gl | 0.119 | [-0.378, 0.616] | 0.928 |
| Gl - Ctrl | 0.094 | [-0.403, 0.591] | 0.968 |

**Table S3:** *Post hoc* comparison test for soil FDA hydrolyzed (μg/mg soil) legacies after conditioning phase across five categories of soil origin at p = 0.05. n_1_ = n_2_ = n_3_ = n_4_ = n_5_ = 3, N = 15. Significant *p* values are < 0.05.
