# Supplementary material for: Plant–soil feedback responses of four dryland crop species under greenhouse conditions
Source: Plant Environ Interact. 2020 Dec 7;1(3):181–95. doi: 10.1002/pei3.10035 (PMC10168064; doi:10.1002/pei3.10035)
Supplement: Supplementary file 7 — Table S4 [file PEI3-1-181-s003.docx]

| Soil origin | Mean difference | Confidence interval | p-value |
| --- | --- | --- | --- |
| He/Ph – Ctrl | 1.298 | [0.327, 2.269] | 0.009 |
| He/Ph – He/Gl | 0.944 | [-0.027, 1.916] | 0.058 |
| He/Ph – He/Ze | 0.521 | [-0.450, 1.492] | 0.441 |
| He/Ph – He/He | 0.718 | [-0.253, 1.689] | 0.183 |
| He/Ze – Ctrl | 0.777 | [0.194, 1.748] | 0.137 |
| He/Ze – He/Gl | 0.424 | [-0.548, 1.395] | 0.621 |
| He/Ze – He/He | 0.197 | [-0.774, 1.168] | 0.959 |
| He/He – Ctrl | 0.580 | [-0.391, 1.551] | 0.346 |
| He/He – He/Gl | 0.226 | [-0.745, 1.198] | 0.934 |
| He/Gl – Ctrl | 0.354 | [-0.618, 1.325] | 0.753 |

**Table S4:** Tukey’s honestly significant difference (Tukey HSD) *post hoc* comparison test for soil FDA hydrolyzed (μg/mg soil) legacies after feedback phase across five categories of soil origin at p = 0.05. n_1_ = n_2_ = n_3_ = n_4_ = n_5_ = 3, N = 15. Significant *p* values are < 0.05.
