# Supplementary material for: Plant–soil feedback responses of four dryland crop species under greenhouse conditions
Source: Plant Environ Interact. 2020 Dec 7;1(3):181–95. doi: 10.1002/pei3.10035 (PMC10168064; doi:10.1002/pei3.10035)
Supplement: Supplementary file 8 — Table S5 [file PEI3-1-181-s004.docx]

| Effect | d.f. | Soil nitrogen | | | | | | | |
| --- | --- | --- | --- | --- | --- | --- | --- | --- | --- |
|  |  | NO_3_ – N | | NH_4_ – N | | NO_3_: NH_4_ ratio | | Total nitrogen | |
|  |  | *H* | p | *H* | p | *H* | p | *H* | p |
| Soil origin | 8 | 25.4 | 0.001 | 18.9 | 0.015 | 25.4 | 0.001 | 24.03 | 0.002 |

**Table S5:** Kruskal-Wallis test for soil nitrogen legacies across nine categories of soil origin at p=0.05. n_1_ = n_2_ = n_3_ = n_4_ = n_5_ = 3, n_6_ = n_7_ = n_8_ = n_9_ = 9, N = 51. Significant *p* values are < 0.05.
