# Supplementary material for: Plant–soil feedback responses of four dryland crop species under greenhouse conditions
Source: Plant Environ Interact. 2020 Dec 7;1(3):181–95. doi: 10.1002/pei3.10035 (PMC10168064; doi:10.1002/pei3.10035)
Supplement: Supplementary file 9 — Table S6 [file PEI3-1-181-s011.docx]

| Feedback effect | Soil nitrogen | | | | | | | |
| --- | --- | --- | --- | --- | --- | --- | --- | --- |
|  | NO_3_ – N | | NH_4_ – N | | NO_3_: NH_4_ ratio | | Total nitrogen | |
|  | *H* | p | *H* | P | *H* | p | *H* | P |
| ^‡^He/^‡^Gl | 6.33 | 1.000 | 1.22 | 1.000 | 7.11 | 1.000 | 6.50 | 1.000 |
| ^‡^He/^₤^He | 11.2 | 1.000 | 19.1 | 1.000 | 22.5 | 0.835 | 12.2 | 1.000 |
| ^‡^He/^‡^Ze | -14.1 | 1.000 | -4.22 | 1.000 | -13.1 | 1.000 | -8.94 | 1.000 |
| ^‡^He/^₤^Gl | 14.6 | 1.000 | 1.78 | 1.000 | 12.2 | 1.000 | 7.50 | 1.000 |
| ^‡^He/^₤^Ph | 19.7 | 1.000 | 16.1 | 1.000 | 15.2 | 1.000 | 19.7 | 1.000 |
| ^‡^He/^‡^Ph | -20.4 | 0.123 | -7.28 | 1.000 | -19.5 | 0.194 | -13.6 | 1.000 |
| ^‡^He/^₤^Ze | 32.7 | 0.033 | 3.22 | 1.000 | 34.8 | 0.016 | 25.8 | 0.328 |
| ^‡^He/Ctrl | 38.1 | 0.004 | 27.6 | 0.191 | 37.2 | 0.006 | 33.5 | 0.026 |
| ^‡^Gl/^₤^He | 4.89 | 1.000 | 20.3 | 1.000 | 15.4 | 1.000 | 18.7 | 1.000 |
| ^‡^Gl/^‡^Ze | -7.78 | 1.000 | -3.00 | 1.000 | -5.94 | 1.000 | -2.44 | 1.000 |
| ^‡^Gl/^₤^Gl | 8.22 | 1.000 | 0.556 | 1.000 | 5.06 | 1.000 | 1.00 | 1.000 |
| ^‡^Gl/^₤^Ph | 13.4 | 1.000 | 14.9 | 1.000 | 8.06 | 1.000 | 13.2 | 1.000 |
| ^‡^Gl/^‡^Ph | -14.1 | 1.000 | -6.06 | 1.000 | -12.4 | 1.000 | -7.11 | 1.000 |
| ^‡^Gl/^₤^Ze | 26.4 | 0.271 | 4.44 | 1.000 | 27.7 | 0.186 | 19.3 | 1.000 |
| ^‡^Gl/Ctrl | 31.7 | 0.047 | 26.4 | 0.278 | 30.1 | 0.087 | 27.0 | 0.231 |
| ^₤^He/^‡^Ze | -2.89 | 1.000 | -23.3 | 0.676 | -9.44 | 1.000 | -21.1 | 1.000 |
| ^₤^He/^₤^Gl | 3.33 | 1.000 | 20.8 | 1.000 | 10.3 | 1.000 | 19.7 | 1.000 |
| ^₤^He/^₤^Ph | -8.50 | 1.000 | -35.2 | 0.135 | -7.33 | 1.000 | -31.8 | 0.313 |
| ^₤^He/^‡^Ph | -9.22 | 1.000 | -26.3 | 0.283 | -3.00 | 1.000 | -25.8 | 0.334 |
| ^₤^He/^₤^Ze | -21.5 | 1.000 | -15.8 | 1.000 | -12.3 | 1.000 | -38.0 | 0.063 |
| ^₤^He/Ctrl | 26.8 | 0.955 | 46.7 | 0.004 | 14.7 | 1.000 | 45.7 | 0.006 |
| ^‡^Ze/^₤^Gl | 0.444 | 1.000 | 2.44 | 1.000 | 0.889 | 1.000 | 1.44 | 1.000 |
| ^‡^Ze/^₤^Ph | 5.61 | 1.000 | 11.9 | 1.000 | 2.11 | 1.000 | 10.7 | 1.000 |
| ^‡^Ze/^‡^Ph | 6.33 | 1.000 | 3.06 | 1.000 | 6.44 | 1.000 | 4.67 | 1.000 |
| ^‡^Ze/^₤^Ze | 18.6 | 1.000 | 7.44 | 1.000 | 21.8 | 1.000 | 16.9 | 1.000 |
| ^‡^Ze/Ctrl | 23.9 | 0.552 | 23.4 | 0.656 | 24.1 | 0.539 | 24.6 | 0.475 |
| ^₤^Gl/^₤^Ph | -5.17 | 1.000 | -14.3 | 1.000 | -3.00 | 1.000 | -12.2 | 1.000 |
| ^₤^Gl/^‡^Ph | -5.89 | 1.000 | -5.50 | 1.000 | -7.33 | 1.000 | -6.11 | 1.000 |
| ^₤^Gl/^₤^Ze | -18.2 | 1.000 | -5.00 | 1.000 | -22.7 | 1.000 | -18.3 | 1.000 |
| ^₤^Gl/Ctrl | 23.5 | 1.000 | 25.8 | 1.000 | 25.0 | 1.000 | 26.0 | 1.000 |
| ^₤^Ph/^‡^Ph | -0.722 | 1.000 | 8.83 | 1.000 | -4.33 | 1.000 | -6.06 | 1.000 |
| ^₤^Ph/^₤^Ze | -13.0 | 1.000 | -19.3 | 1.000 | -19.7 | 1.000 | -6.17 | 1.000 |
| ^₤^Ph/Ctrl | 18.3 | 1.000 | 11.5 | 1.000 | 22.0 | 1.000 | 13.8 | 1.000 |
| ^‡^Ph/^₤^Ze | 12.3 | 1.000 | 10.5 | 1.000 | 15.3 | 1.000 | 12.2 | 1.000 |
| ^‡^Ph/Ctrl | 17.6 | 1.000 | 20.3 | 1.000 | 17.7 | 1.000 | 19.9 | 1.000 |
| ^₤^Ze/Ctrl | 5.33 | 1.000 | 30.8 | 0.398 | 2.33 | 1.000 | 7.67 | 1.000 |

**Table S6:** Kruskal-Wallis pairwise comparisons for soil nitrogen legacies across plant-soil feedback procedures at p = 0.05. ^‡^ Heterospecifics, ^₤^ Conspecifics, Ctrl Procedure Control. Coefficient of variation (CV) in soil nitrogen were; 2.3 %, 0.4 %, 1.4 % and 1.5 % for NO_3_ – N, NH_4_ – N, Total nitrogen and NO_3_: NH_4_ ratio, respectively. Significant *p* values are < 0.05.
