# Supplementary material for: Plant–soil feedback responses of four dryland crop species under greenhouse conditions
Source: Plant Environ Interact. 2020 Dec 7;1(3):181–95. doi: 10.1002/pei3.10035 (PMC10168064; doi:10.1002/pei3.10035)
Supplement: Supplementary file 10 — Table S7 [file PEI3-1-181-s005.docx]

| Soil origin | Total GP bacteria | Total GN bacteria | GP to GN ratio | Total bacterial PLFAs | Total fungal PLFAs | Fungal to bacterial ratio |
| --- | --- | --- | --- | --- | --- | --- |
| Phase 1 | | | | | | |
| Ze | 35.30 | 411.51 | 0.086 | 534.25 | 664.44 | 1.24 |
| Ph | 20.73 | 384.64 | 0.054 | 490.01 | 606.19 | 1.24 |
| He | 11.97 | 382.30 | 0.031 | 462.65 | 607.22 | 1.31 |
| Gl | 23.12 | 382.27 | 0.060 | 475.26 | 606.19 | 1.28 |
| Ctrl | 34.46 | 830.98 | 0.041 | 939.52 | 551.10 | 0.59 |
| Phase 2 | | | | | | |
| He/Ze | 25.68 | 132.17 | 0.19 | 192.86 | 110.95 | 0.58 |
| He/He | 0 | 135.11 | 0.00 | 160.13 | 191.97 | 1.20 |
| He/Gl | 0 | 128.98 | 0.00 | 162.62 | 123.49 | 0.76 |
| He/Ph | 0 | 129.90 | 0.00 | 152.87 | 93.90 | 0.61 |
| Ctrl | 0 | 131.49 | 0.00 | 162.76 | 107.31 | 0.66 |

**Table S7:** Total abundance (nmol/g) and ratios of viable microbes present in soil of different origin at the end of phase 1 monocultures and phase 2 rotations using fungal and bacterial PLFA signatures. Ctrl – procedural bare control soil, Gl – *G. max*, He – *H. annuus*, Ph – *P. vulgaris*, and Ze – *Z. mays*, He/Ze heterospecific *Z. mays* feedback, He/He conspecific *H. annuus* feedback, He/Ph heterospecific *P. vulgaris* feedback and He/Gl heterospecific *G. max* feedback, GN – Gram-negative bacteria and GP – Gram-positive bacteria.
