# Supplementary material for: Plant–soil feedback responses of four dryland crop species under greenhouse conditions
Source: Plant Environ Interact. 2020 Dec 7;1(3):181–95. doi: 10.1002/pei3.10035 (PMC10168064; doi:10.1002/pei3.10035)
Supplement: Supplementary file 11 — Table S8 [file PEI3-1-181-s009.docx]

| Soil origin | C18:2ω6c | C18:1ω9t | C18:1ω9c | C18:2ω6t | C18:3ω3c | C14:0 | i-C15:0 | a-C15:0 | C15:0 | i-C16:0 | C16:19 | C17:0 | i-C17:0 | C18:17 |
| --- | --- | --- | --- | --- | --- | --- | --- | --- | --- | --- | --- | --- | --- | --- |
| P1 | Fungal fatty acids biomarkers | | | | | Bacterial fatty acids biomarkers | | | | | | | | |
| Ze | 122.61 | 10.71 | 372.12 | 145.28 | 13.72 | 62.93 | 25.29 | 0 | 12.33 | 10.01 | 411.51 | 12.18 | 0 | 0 |
| Ph | 110.75 | 9.56 | 331.36 | 138.74 | 15.88 | 62.88 | 8.71 | 0 | 11.15 | 12.02 | 384.64 | 10.61 | 0 | 0 |
| He | 98.03 | 5.59 | 360.30 | 135.47 | 7.83 | 55.32 | 6.76 | 0 | 9.02 | 5.21 | 382.30 | 4.04 | 0 | 0 |
| Gl | 109.24 | 10.15 | 340.33 | 137.72 | 8.76 | 56.01 | 10.47 | 0 | 9.70 | 12.65 | 382.27 | 4.17 | 0 | 0 |
| Ctrl | 95.90 | 9.77 | 301.26 | 137.38 | 6.78 | 59.10 | 22.46 | 0 | 14.98 | 12.00 | 830.98 | 0 | 0 | 0 |
| P2 | Fungal fatty acids biomarkers | | | | | Bacterial fatty acids biomarkers | | | | | | | | |
| He/Ze | 8.92 | 0 | 29.07 | 72.97 | 0 | 2.53 | 12.71 | 12.97 | 0 | 0 | 132.17 | 0 | 0 | 32.47 |
| He/He | 78.90 | 0 | 27.33 | 73.86 | 11.89 | 0 | 0 | 0 | 0 | 0 | 135.11 | 0 | 0 | 25.02 |
| He/Gl | 6.53 | 0 | 31.14 | 85.82 | 0 | 9.64 | 0 | 0 | 0 | 0 | 128.98 | 0 | 0 | 24.00 |
| He/Ph | 7.28 | 0 | 17.01 | 69.61 | 0 | 0 | 0 | 0 | 0 | 0 | 129.90 | 0 | 0 | 22.96 |
| Ctrl | 6.74 | 0 | 17.35 | 83.22 | 0 | 12.24 | 0 | 0 | 0 | 0 | 131.49 | 0 | 0 | 19.02 |

**Table S8:** PLFA biomarkers in phases 1 (P1) and 2 (P2).
